# Altered organization of the intermediate filament cytoskeleton and relocalization of proteostasis modulators in cells lacking the ataxia protein sacsin

**DOI:** 10.1093/hmg/ddx197

**Published:** 2017-05-23

**Authors:** Emma J. Duncan, Roxanne Larivière, Teisha Y. Bradshaw, Fabiana Longo, Nicolas Sgarioto, Matthew J. Hayes, Lisa E.L. Romano, Suran Nethisinghe, Paola Giunti, Michaela B. Bruntraeger, Heather D. Durham, Bernard Brais, Francesca Maltecca, Benoit J. Gentil, J. Paul Chapple

**Affiliations:** 1 William Harvey Research Institute, Barts and the London School of Medicine, Queen Mary University of London, London EC1M 6BQ, UK; 2 Department of Neurology and Neurosurgery, Montreal Neurological Institute, McGill University, Montreal, QC H3A 2B4, Canada; 3 Università Vita-Salute San Raffaele and Division of Genetics and Cell Biology, San Raffaele Scientific Institute, Milan, Italy; 4 Università degli Studi dell’Insubria, Varese, Italy; 5 UCL Institute of Ophthalmology, London EC1V 9E, UK; 6 Department of Molecular Neuroscience, UCL Institute of Neurology, London WC1N 3BG, UK; 7 Laboratory of Neurogenetics of Motion, Montreal Neurological Institute, McGill University, Montreal, QC H3A 2B4, Canada

## Abstract

Autosomal Recessive Spastic Ataxia of Charlevoix-Saguenay (ARSACS) is caused by mutations in the gene *SACS*, encoding the 520 kDa protein sacsin. Although sacsin’s physiological role is largely unknown, its sequence domains suggest a molecular chaperone or protein quality control function. Consequences of its loss include neurofilament network abnormalities, specifically accumulation and bundling of perikaryal and dendritic neurofilaments. To investigate if loss of sacsin affects intermediate filaments more generally, the distribution of vimentin was analysed in ARSACS patient fibroblasts and in cells where sacsin expression was reduced. Abnormal perinuclear accumulation of vimentin filaments, which sometimes had a cage-like appearance, occurred in sacsin-deficient cells. Mitochondria and other organelles were displaced to the periphery of vimentin accumulations. Reorganization of the vimentin network occurs *in vitro* under stress conditions, including when misfolded proteins accumulate. In ARSACS patient fibroblasts HSP70, ubiquitin and the autophagy-lysosome pathway proteins Lamp2 and p62 relocalized to the area of the vimentin accumulation. There was no overall increase in ubiquitinated proteins, suggesting the ubiquitin–proteasome system was not impaired. There was evidence for alterations in the autophagy–lysosome pathway. Specifically, in ARSACS HDFs cellular levels of Lamp2 were elevated while levels of p62, which is degraded in autophagy, were decreased. Moreover, autophagic flux was increased in ARSACS HDFs under starvation conditions. These data show that loss of sacsin effects the organization of intermediate filaments in multiple cell types, which impacts the cellular distribution of other organelles and influences autophagic activity.

## Introduction

Autosomal Recessive Spastic Ataxia of Charlevoix-Saguenay (ARSACS) is a neurodegenerative disease associated with progressive loss of Purkinje neurons (1,2). It is a childhood onset condition that is characterized by cerebellar ataxia, pyramidal spasticity and peripheral neuropathy. At the genetic level, ARSACS is caused by mutations in the *SACS* gene (3). This encodes the extremely large (4579 amino acid) modular protein sacsin, which from its N- to C-terminus is composed of a ubiquitin-like domain that binds to the proteasome (4), three large sacsin repeat regions that may have an Hsp90-like function (5,6), a J-domain that binds HSP70 (4,5) and a higher eukaryotes and prokaryotes nucleotide-binding domain that can dimerise (7). Based on the presence of these conserved domains, some of which are present in molecular chaperones and components of the ubiquitin–proteasome system, it is a possibility that sacsin may function in proteostasis.

It is unclear if a molecular chaperone role for sacsin would be consistent with findings from cellular and mouse models of ARSACS, where cytoskeletal and mitochondrial abnormalities have been identified. Specifically, in the *Sacs*^−^^/^^−^ mouse we have shown that loss of sacsin results in abnormal neurofilament accumulations in the somatodendritic regions of several neuronal populations as early as 14 days after birth (8). Furthermore, in cultured hippocampal neurons where sacsin was targeted with shRNA, and in spinal motor neurons and dorsal root ganglion (DRG) neurons cultured from *Sacs*^*−*^^*/*^^*−*^ mice, a similar redistribution of neurofilament was observed. These abnormal neurofilament accumulations were demonstrated to contain the hypo-phosphorylated form of neurofilament heavy chain protein (NFH) (8). In addition to intermediate filament defects, loss of sacsin altered mitochondrial morphology, dynamics and distribution. Mitochondrial length is increased (2,8,9), consistent with reduced mitochondrial recruitment of the fission factor dynamin related protein 1 (Drp1) contributing to this phenotype (9). In agreement with others, we have also demonstrated that the morphological alterations in mitochondrial networks are accompanied by impaired oxidative phosphorylation and increased oxidative stress (2,9,10). Mitochondrial motility was impaired in motor neurons cultured from *Sacs*^−^^/^^−^ mice (8). Together these data indicate that loss of sacsin causes problems with both the intermediate filament cytoskeleton and mitochondria function, phenotypic aspects that are not mutually exclusive given the importance of the cytoskeleton in regulating mitochondrial dynamics (11,12).

As we had observed impaired mitochondrial function in human dermal fibroblasts (HDFs) from ARSACS patients (2,9,10), we hypothesized that these cells may also exhibit cytoskeletal changes. Thus, we investigated the organization of the vimentin intermediate filament network in HDFs derived from skin biopsies of ARSACS patients (vimentin being the major intermediate filament protein in fibroblasts). In a proportion of ARSACS cells, intermediate filaments were bundled and collapsed as a cage-like structure around the microtubule organizing centre (MTOC) rather than radiating from the nucleus towards the plasma membrane, as in control cells. These vimentin structures were reminiscent of the ‘cage’ of the vimentin intermediate filaments that surround aggresomes. Aggresomes form when misfolded protein aggregates are trafficked in a retrograde manner to the MTOC, where they accumulate prior to clearance by the autophagy–lysosome machinery (13,14). Thus, we investigated the distribution and function of the proteostasis machinery in ARSACS fibroblasts, observing components of the cellular proteostasis machinery relocalized within the accumulations of vimentin. Moreover, aspects of the phenotype were recapitulated in primary neurons from the *Sacs*^−^^/^^−^ mouse. In combination, our data show that loss of sacsin impacts on intermediate filament organization leading to changes in the cellular distribution of proteostasis system components and altered autophagy.

## Results

### The vimentin intermediate filament cytoskeleton is disrupted in ARSACS patient fibroblasts

We primarily report on ARSACS HDF lines from four patients that are compound heterozygous (p.Q4054* & c.2094-2A > C; p.R2002fs & p.Q4054*; p.F2780C & p.S3892*; p.K1715* & p.R4331Q) and one patient that is homozygous (p.2801delQ) (Supplementary Material, Fig. S1A). Cell lines from two further compound heterozygous patients, sharing two variants in cis on one allele (p.R3636Q:p.P3652T) and a different frameshift on the other allele (p.L3745Rfs or p.C72Cfs) (15), plus two patients homozygous for the c.8844delT mutation, the major founder mutation in Québec, were also investigated (Supplementary Material, Figs S1C and D). Using an antibody directed to the C-terminus of sacsin, immunoblot showed that levels of the protein were similar in control HDFs and the HDF line with the p.2801delQ mutation. Some sacsin was also detected in HDFs from the ARSACS patient with the p.R3636Q:p.P3652T:p.C72Cfs mutations (Supplementary Material, Fig. S1). Sacsin was not conclusively detected in cell lines from patients with the other mutations investigated. 

**Figure 1:**
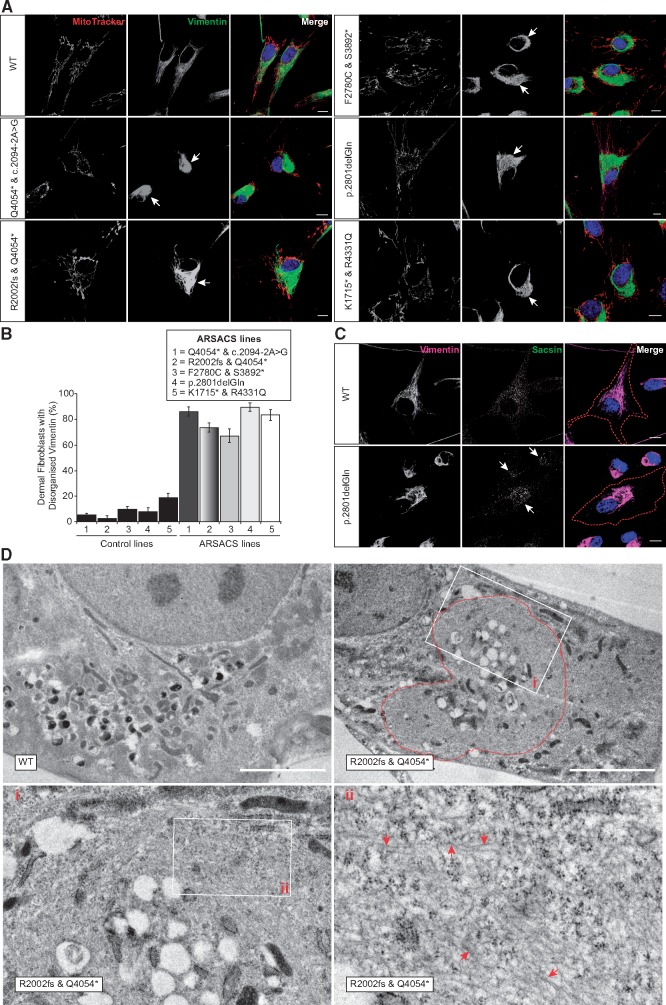
Abnormal accumulations of vimentin intermediate filament in ARSACS patient HDFs. (**A**) Representative confocal images of five ARSACS patient HDFs and a wild-type (WT) control HDF line (further patient and control lines are shown in Supplementary Material, Fig. S2) that were stained for mitochondria (MitoTracker) and immunolabelled for vimentin. Cells were also stained with DAPI to detect nuclei. Arrows indicate areas of abnormal perinuclear vimentin accumulation. Scale bars = 10 μm. (**B**) The percentage of cells with a collapsed vimentin network was then quantified for each cell line. This was done blind to experimental status with >120 cells scored per cell line. Results are expressed as mean±SEM for each control and patient cell line. (**C**) Representative confocal images of HDFs from a homozygous ARSACS patient with the mutation p.2801delQ and a wild-type control line. HDFs were processed for immunofluorescent detection of sacsin and vimentin, and were counterstained with DAPI for nuclei. Localization of sacsin to the area of perinuclear vimentin accumulation is indicated by an arrow. Red dotted line indicates the edge of the cell. Scale bars = 10 μm. (**D**) Representative TEM of a HDF cell from a wild-type control and ARSACS patient (p.R2002fs & p.Q4054*). Boxes delineated by white lines indicate position of zoomed images shown in panels I and II. Red dotted line indicates the approximate boundary of the area of accumulation of filamentous material. Examples of filaments are indicated with arrows. Scale bar = 5 μm.

Confocal imaging revealed that a large proportion of cells in all of the ARSACS patients HDF lines investigated had a grossly abnormal vimentin cytoskeleton. In contrast to wild-type control cells, where vimentin was distributed throughout the cytoplasm, vimentin filaments were bundled in perinuclear accumulations in patient cells (Fig. 1A and B and Supplementary Material, Fig. S2). This phenotype was highly penetrant with 79.6 ± 1.9% of ARSACS HDFs (Fig. 1B and Supplementary Material, Fig. S2A) having a disorganized vimentin network, relative to just 8.5 ± 1.2% of control cells. Vimentin filaments were also disrupted in the ARSACS lines that still have detectable sacsin protein, with sacsin being localized to the area of filament accumulation, rather than being distributed throughout the cytoplasm, as in wild-type control cells (Fig. 1C).

Transmission electron microscopy (TEM) revealed that in ARSACS HDFs cytoplasmic organelles were localized peripherally to a perinuclear region of uniform electron density (Fig. 1D). Higher-magnification imaging indicated this region contained significant amounts of ∼10 nm filamentous material, consistent with the size of intermediate filaments. The region of filamentous material accumulation frequently contained vesicular structures and vacuoles.

### Sacsin knockdown disrupts vimentin intermediate filament organization in wild-type cells

**Figure 2:**
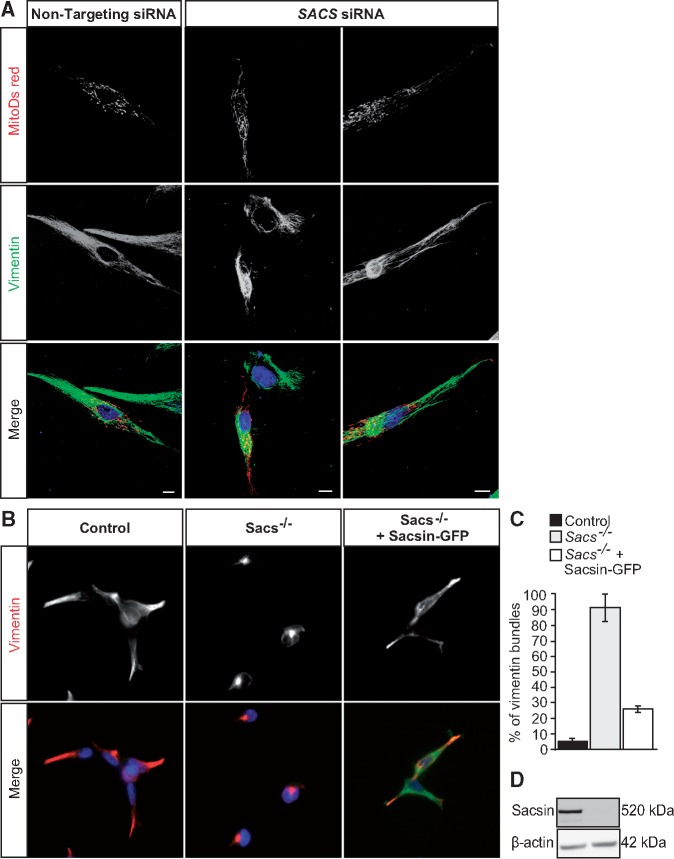
Abnormal accumulations of vimentin intermediate filament in sacsin knockdown cells. (**A**) Representative confocal images of wild-type HDFs cotransfected with mitoDSRed and either a non-targeting siRNA or siRNA targeting sacsin (*SACS)*. 48 h after transfection, cells were processed for immunofluorescent detection of vimentin and counterstained with DAPI for nuclei. Scale bars = 10 µm. (**B**) Representative confocal images of CRISPR generated *SACS*^−/−^ HEK293 cells and *SACS*^−/−^ HEK293 transfected with a plasmid for expression of full-length sacsin-GFP. Cells were processed for immunofluorescent detection of vimentin and counterstained with DAPI. (**C**) The percentage of cells with a collapsed vimentin network was then quantified for each condition. This was done blind to experimental status with >45 cells scored per condition. Results are expressed as mean ± SEM. (**D**) Sacsin immunoblot of total lysates from control and *SACS*^−/−^ HEK293 cells. β-actin was used as a loading control.

To further confirm that loss of functional sacsin results in altered vimentin organization, siRNA-mediated knockdown was used to deplete sacsin in wild-type HDFs, using previously validated siRNAs (4,9). A significant increase (*P* < 0.01) in the percentage of sacsin knockdown HDFs that exhibited a perinuclear accumulation of vimentin was observed relative to controls, with vimentin bundles visible in some cells (Fig. 2A). We also made a HEK293 sacsin knockout line using CRISPR/Cas9 genome editing. These cells did not express detectable levels of sacsin and also exhibited perinuclear collapse of the vimentin network (Fig. 2B and C). In these cells, we were able to rescue the vimentin phenotype by transfection of a plasmid for expression of full-length sacsin-GFP (Fig. 2B and C).

### Vimentin accumulates around the MTOC and disrupts the distribution of organelles in ARSACS patient cells

**Figure 3:**
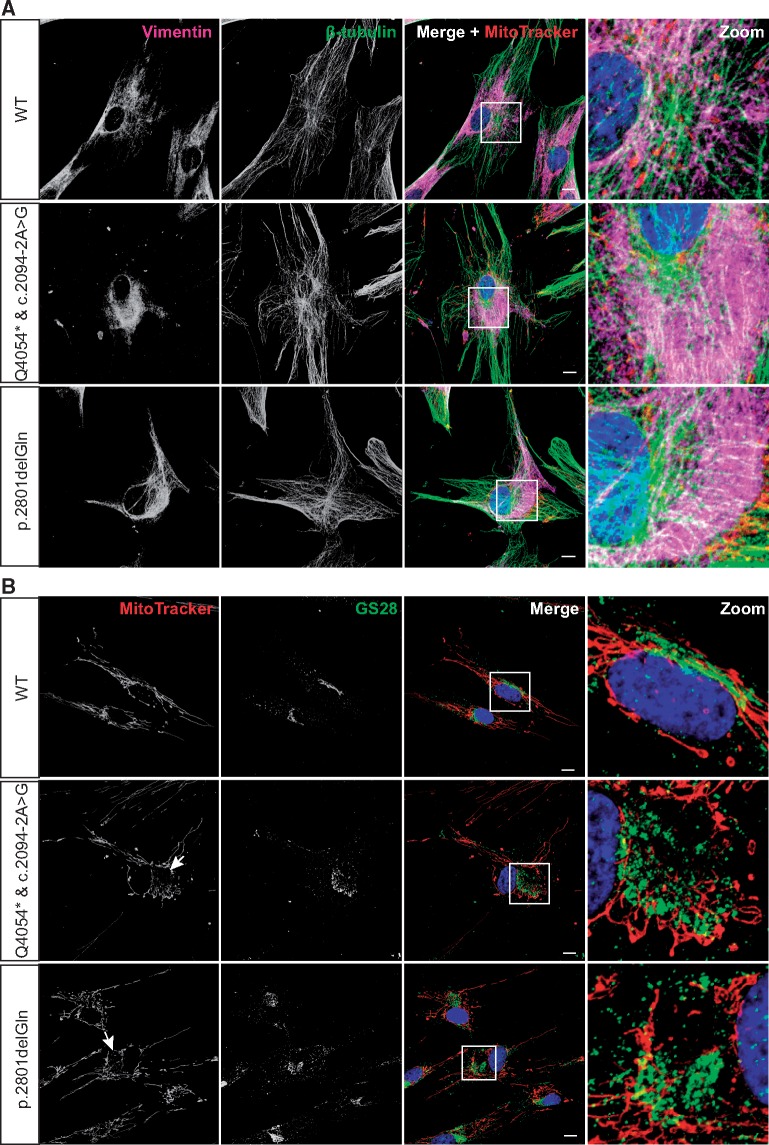
ARSACS HDFs show characteristics of aggresome formations, including accumulation of vimentin around the MTOC and Golgi fragmentation. (**A,B**) Representative confocal images of two ARSACS patient HDFs lines (heterozygous mutations c.2094-2A > G/Q4054* and the homozygous mutation p.2801delQ) and a WT control (further patient and control lines are shown in Supplementary Material, Fig. S3). Cell were stained with MitoTracker before being processed for immunofluorescent detection of (**A**) vimentin and β-tubulin, or (**B**) GS28, a membrane protein of the *cis*-Golgi. Cells were counterstained with DAPI for nuclei. White boxes are shown as zoomed images in the right-hand panels. Arrows indicate areas of mitochondrial network disruption. Scale bars = 10 μm.

As cytoskeletal elements are interlinked we used confocal analyses to investigate if the actin filament and microtubule networks were disrupted in ARSACS HDFs. We observed no gross abnormalities in actin microfilament (not shown) or microtubule organization. However, labelling with anti-tubulin did reveal that the abnormal accumulations of vimentin formed in close proximity to the MTOC (Fig. 3A and Supplementary Material, Fig. S3A).

Collapse of the vimentin network would be expected to have consequences for the distribution of organelles, as the cytoskeleton organizes the intracellular space. Indeed, confocal imaging showed displacement of mitochondria to areas peripheral to the abnormal accumulations of vimentin in ARSACS patient cells (Fig. 1A). Disruption of the intermediate filament cytoskeleton has also been reported to interfere with Golgi organization (16,17). Confocal imaging of HDFs immunolabelled with the Golgi protein, GS28, revealed a fragmented Golgi stack, with cisternae displaced around an area of cytoplasm where mitochondria where also absent (Fig. 3B and Supplementary Material, Fig. S3B). With available antibodies, it was not possible to double-label cells with antibodies to vimentin and GS28, but it is likely that fragmented Golgi was displaced around areas of vimentin accumulation. Distribution of the endosome marker, EEA1, was also altered in ARSACS HDFs. Relative to wild-type controls, endosomes were less evenly distributed throughout the cell (Supplementary Material, Fig. S3C), distributing with displaced mitochondria.

### Components of the cellular proteostasis machinery relocalize to the region of abnormal vimentin accumulation in ARSACS patient cells

**Figure 4:**
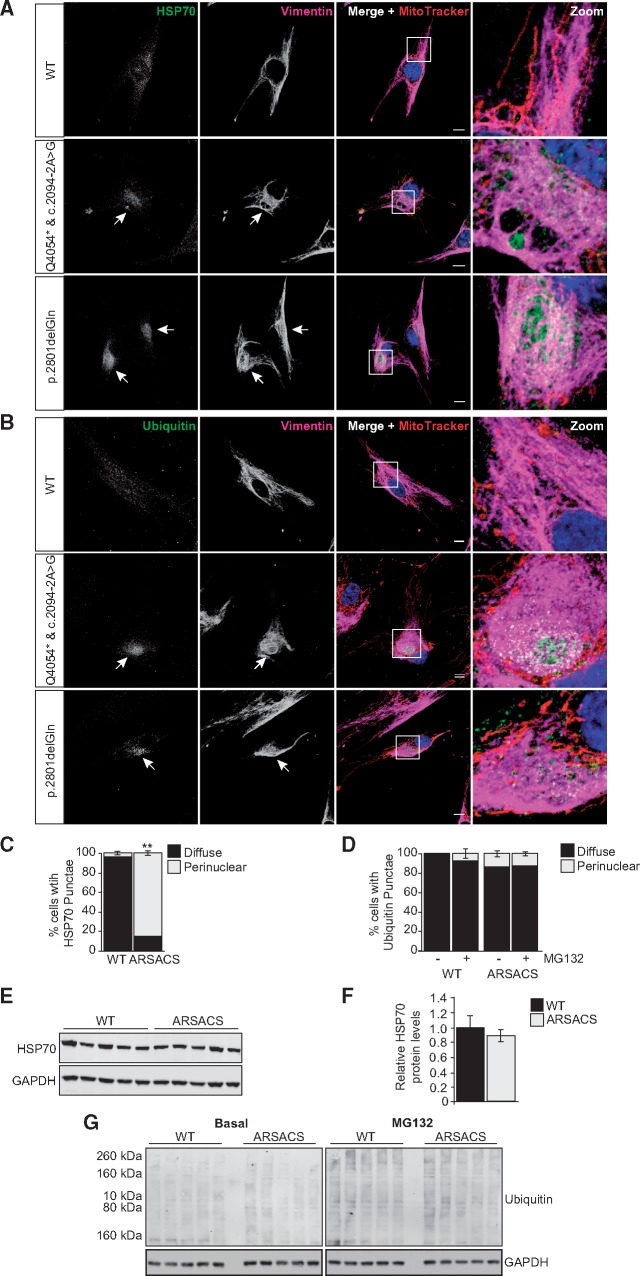
Components of the cellular proteostasis machinery localise to the vimentin cage that forms in ARSACS patient HDFs. (**A,B**) Representative confocal images of two ARSACS patient HDFs lines (heterozygous mutations c.2094-2A > G/p.Q4054* and the homozygous mutation p.2801delQ) and a WT control (further patient and control lines are shown in Supplementary Material, Fig. S4). Cells were immunolabelled for **(**A**)** HSP70 and vimentin, or **(**B**)** ubiquitin and vimentin, as well as mitochondria (MitoTracker) and nuclei (DAPI). White arrows indicate perinuclear accumulation of HSP70 or ubiquitin. White boxes in merged panels are shown zoomed. Scale bars = 10 μm. (**C,D**) The incidence of cells with perinulear localization of (**C**) HSP70 or (**D**) ubiquitin was quantified. For ubiquitin, quantification was performed in cells cultured under control conditions (vehicle only) or treated with the proteasome inhibitor MG132 for 3 h. Results are expressed as mean ± SEM. **(E)** Immunoblot analysis of total cell lysates from five ARSACS patient and five WT control HDFs probed with an anti-HSP70 antibody. GAPDH was used as a loading control. (**F**) Densitometric analyses were performed and mean relative HSP70 protein levels calculated for the five WT and five patient cell lines. Data were normalized to GAPDH. **(G)** Immunoblot analysis of total cell lysates from five ARSACS patient and five WT control HDFs cultured for 3 h in the presence of MG132 or vehicle only control, probed with an anti-ubiquitin antibody. GAPDH was used as a loading control.

The combination of perinuclear accumulation of vimentin around the MTOC, disrupted mitochondrial network organization and Golgi fragmentation have been linked to aggresome formation in cultured cells. Aggresomes are juxtanuclear accumulations of misfolded proteins that form through a regulated process when cellular protein-degradation systems become overwhelmed, a common phenomenon in neurodegenerative disorders (18–20). To look for evidence of disrupted proteostasis in ARSACS HDFs, cells were immunolabelled with an antibody that detects both constitutive (HSPA8) and inducible (HSPA1) isoforms of the molecular chaperone HSP70, which interacts with misfolded proteins and can localize to aggresomes (21,22). In control HDFs, HSP70 labelling was distributed throughout the cytoplasm, while in patient cells HSP70 was frequently enriched in the region with abnormal accumulation of vimentin (Fig. 4A and Supplementary Material, Fig. S4A). Confocal z-stack images indicated HSP70 was surrounded by a ‘cage-like’ bundle of vimentin, with some HSP70 also interspersed within the abnormal accumulation of vimentin. HSP70 labelling appeared reduced in other regions of the cytoplasm. This relocalization of HSP70 in ARSACS HDFs was not accompanied by an increase in overall cellular levels of HSPA8 or expression of HSPA1, as determined by immunoblot (Fig. 4E and F).

Misfolded proteins that cannot be refolded by chaperones are normally targeted for degradation by ubiquitination. Thus, we next compared localization of ubiquitin in wild-type and ARSACS HDFs. Similar to HSP70, we observed ARSACS HDFs where ubiquitin labelling was redistributed to the area of vimentin accumulations, rather than being dispersed as in control cells (Fig. 4B). The percentage of cells with this ubiquitin phenotype (14 ± 3%) was low relative to the those that relocalized HSP70 (85 ± 2%) and there was no significant change in level of ubiquitinated proteins in ARSACS HDFs relative to wild-type HDFs. Immunoblots indicated this was the case, both in cells cultured under normal conditions and in cells treated with MG132 to inhibit proteasome activity (Fig. 4G). Together these data indicate that although proteostasis-linked proteins are relocalized in response to loss of sacsin, the ubiquitin–proteasome system does not appear compromised.

### Lamp2 and other components of the autophagy–lysosome pathway are relocalized to the region of abnormal vimentin accumulation in ARSACS patient cells and have altered cellular levels

**Figure 5:**
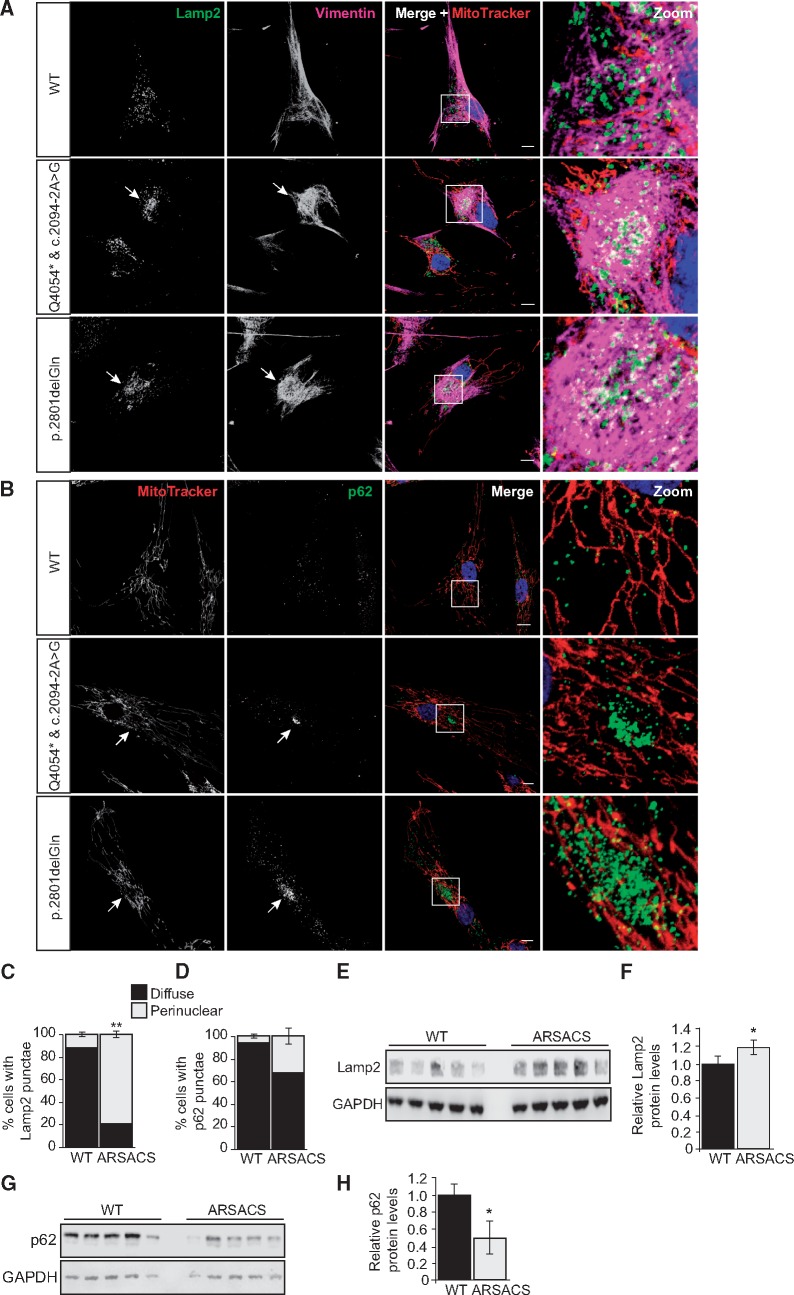
Components of the autophagy–lysosome pathway localise to the vimentin cage that forms in ARSACS patient HDFs. (**A,B**) Representative confocal images of two ARSACS patient HDFs lines (heterozygous mutations c.2094-2A > G/p.Q4054* and the homozygous mutation p.2801delQ) and a WT control (further patient and control lines are shown in Supplementary Material, Fig. S5). Cells were stained for mitochondria (MitoTracker) and then immunolabelled for **(A**) LAMP-2 (lysosome-associated membrane protein 2) and vimentin, or (**B**) p62/SQSTM1. Cells were also stained with DAPI to detect nuclei. White boxes in the merged panels are shown zoomed in the right-hand panels. Arrows indicate areas of LAMP-2 or p62 accumulation. Scale bars = 10 μm. (**C,D**) The incidence of cells with perinulear localization of **(C**) Lamp2 or **(D)** p62 was quantified. **(E,F**) Immunoblot of total cell lysates from five ARSACS patient and five WT control HDFs probed with an anti-Lamp2 antibody and subsequent densitometric analyses. **(G,H**) Immunoblot of total cell lysates from five ARSACS patient and five WT control HDFs probed with an anti-p62 antibody and subsequent densitometric analyses. Data were normalised to GAPDH.

Separate to aggresome formation, vimentin deficiency is associated with juxtanuclear accumulation of lysosomes (23). To ascertain if lysosome localization was altered in ARSACS HDFs, control and patient cells were immunolabelled for vimentin and lysosomal-associated membrane protein 2 (Lamp2). Lamp2 was distributed throughout the cytoplasm in control HDFs. In a significant (*P* < 0.01) percentage of patient cells Lamp2 positive puncta were clustered within the region of vimentin accumulation, here it was both interspersed with the vimentin and localized within the vimentin ‘cage’ (Fig. 5A and C, Supplementary Material, Fig. S5). Similar to Lamp2, the ubiquitin-binding autophagic adaptor protein p62 (also known as SQSTM1), which is required for autophagic clearance of ubiquitinated proteins (24,25), was also relocalized within the vimentin cage (Fig. 5B and D). Immunoblot showed a small, but statistically significant, increase in Lamp2 levels in ARSACS HDF relative to controls (1.19-fold increase, *P* < 0.05) (Fig. 5E and F). Contrastingly, levels of p62 were reduced in ARSACS HDFs relative to controls (0.5-fold decrease, *P* < 0.05). This was of particular interest as p62 has been identified as one of the specific substrates that is degraded through the autophagy–lysosome pathway (26–28).

### Autophagic flux is altered in ARSACS patient cells

**Figure 6:**
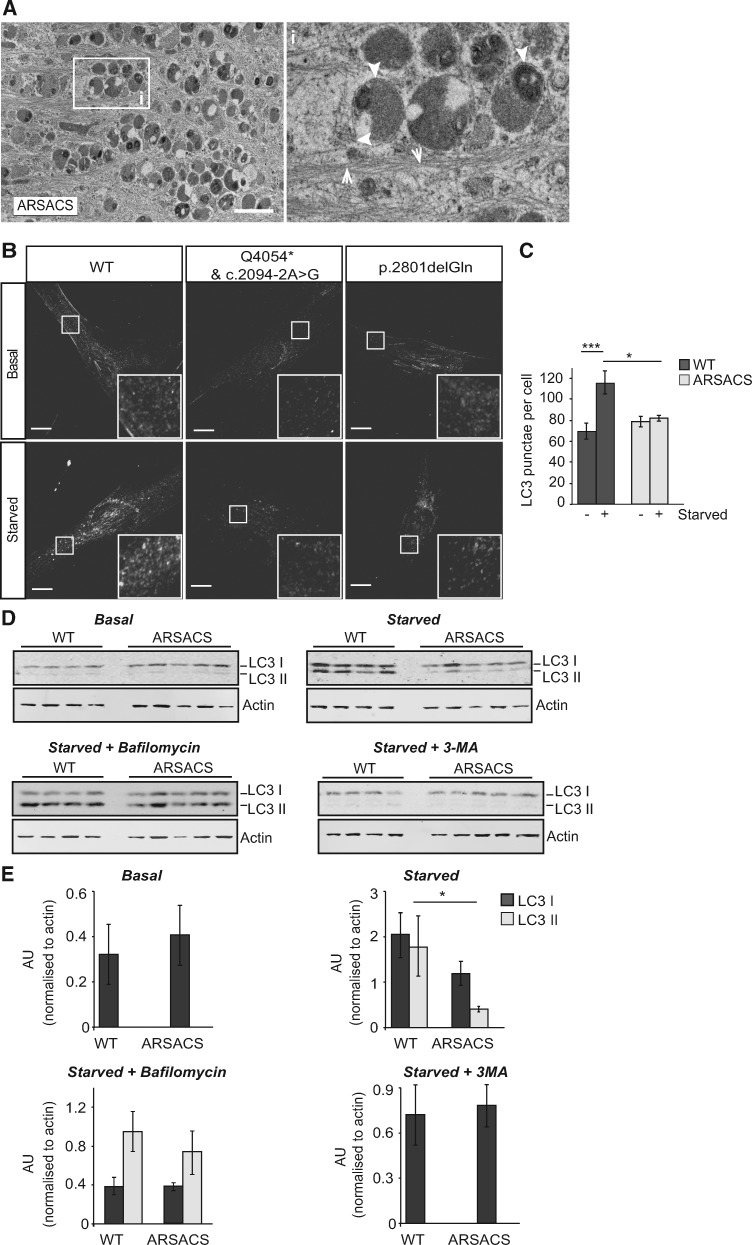
Autopahgic Flux is increased in ARSACS patient HDFs upon nutrient starvation. (**A**) Representative EM image of autophagosomes (arrowheads) in the area of intermediate filament (arrows) accumulation in an ARSACS HDF. Scale bar = 1 μm. (**B**) Representative confocal images of two ARSACS patient HDFs lines (heterozygous mutations c.2094-2A>G/p.Q4054* and the homozygous mutation p.2801delQ) and a WT control immunolabelled for endogenous LC3. Scale bar = 10 μm. (**C**) Quantification of the number of LC3 puncta in ARSACS and control HDF lines under basal conditions and after induction of autophagy by nutrient starvation. Puncta were quantified in 12 cells per line/treatment. Results from five control and five patient cell lines were combined to give an overall mean±SEM, ***P<0.001. (**D**) Immunoblot analysis of total cell lysates from ARSACS patient and WT control HDFs probed with an LC3 antibody to detect LC3-I and LC3-II. Cell lysates were collected from untreated cells, nutrient starved cells, and cells that were nutrient starved and treated with either bafilomycin A or 3-Methyladenine (3-MA). Actin was used as a loading control. (**E**) Densitometric analyses were performed and mean LC3-I and LC3-II levels relative to actin were calculated for each treatment in control and ARSACS HDFs (*n*= 4).

We next considered whether autophagosomes accumulated in the region of the vimentin cage. Ultrastructural analyses of ARSACS patient cells reveals accumulation of filamentous material are interspersed with components of the autophagy–lysosome system, including autophagosomes (Fig. 6A). The presence of autophagosomes within the filament accumulations and the immunolabelling of lysosomes within the vimentin ‘cage’ (Fig. 5A) are consistent with autophagic clearance happening at this location.

To ascertain if there was increased activation of the autophagy-lysosome pathway in ARSACS HDFs, the number of LC3 positive puncta were quantified from confocal images of immuolabelled patient and control cells (Fig. 6B and C). The number of LC3 positive puncta was not significantly different between ARSACS HDFs and control cells under basal conditions. In contrast, induction of autophagy by nutrient starvation only resulted in an increase in LC3 positive puncta in control cells (*P* < 0.001). This indicates that either LC3 positive puncta are not forming in response to nutrient starvation in ARSACS HDFs or their turnover is increased. Since generation of LC3-II from its precursor, LC3-I, correlates with autophagosome formation, levels of LC3-I and LC3-II were assessed by immunoblotting (Fig. 6D and E). Under basal conditions, levels of LC3-I were similar in ARSACS patient and controls, but LC3-II was not detectable at quantifiable levels. When autophagy was induced by nutrient starvation LC3-II levels increased in starved cells, and importantly, significantly more LC3-II accumulated in controls than patient cells. To understand why less LC3-II accumulated in ARSACS HDFs relative to controls, we inhibited lysosomal degradation using bafilomycin A. In nutrient starved cells treated with bafilomycin A, levels of LC3-II were not significantly different between ARSACS and control HDFs. The same result was observed when autophagy was inhibited with 3-Methyladenine (Fig. 6D and E). These data suggest that LC3 is more rapidly degraded and that autophagic flux is increased under conditions that induce autophagy in ARSACS HDFs.

### Neurofilament bundling, organelle displacement and relocalization of ubiquitin occurs in *Sacs*^−^^/^^−^ primary neurons

**Figure 7:**
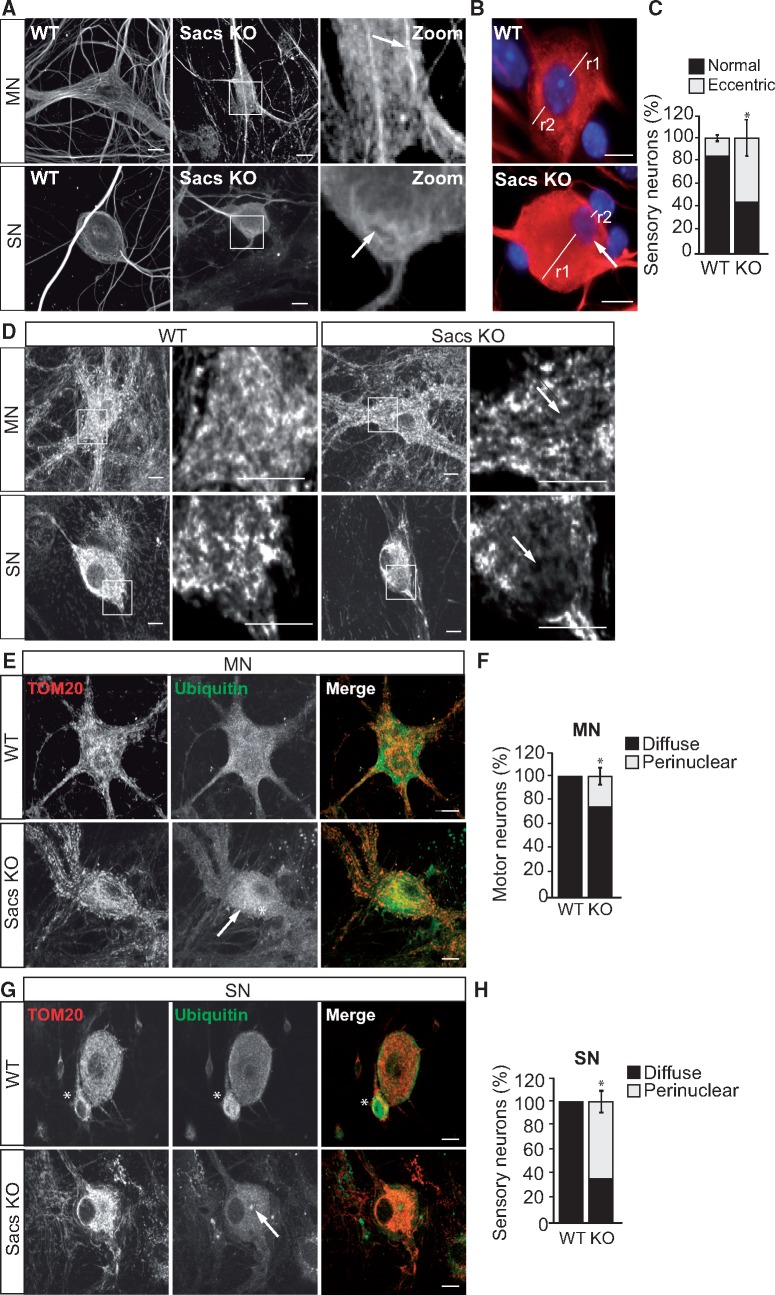
Primary neurons from the *Sacs*^−/−^ mouse have abnormal neurofilament organization, altered cellular architecture and abnormal ubiquitin localization. (**A**) Representative maximum intensity projections of confocal Z-stacks of primary neurons from 4-week-old dorsal root ganglia and spinal cord culture. Motor (MN) and sensory neurons (SN) from *Sacs^−/−^* (Sacs KO) or WT mice were immunolabelled for NFH. Arrows indicate bundled NFH intermediate filaments. (**B**) Nuclear positioning in DRG sensory neurons revealed by DAPI (blue) staining for the nucleus and immunostaining for tubulin (red) to identify the soma in the *Sacs*^−/−^ sensory neuron. (**C**) Quantification of the percentage of sensory neuron with eccentric nuclear localization. Eccentric localization of nucleus was determined by the ratio of r1:r2, where r1 is the longest and r2 is the shortest distance between the nuclear membrane and the closest plasma membrane. Cells where r1/r2 was ≥1.1 (≥10% deviation from a centrally positioned nucleus) were scored as having an eccentric nuclear localization **(D**) Representative confocal images of motor (MN) and sensory neurons (SN) from *Sacs^−/−^* (Sacs KO) or WT mice were immunolabelled for Tom20. Arrows indicate areas where mitochondria were absent. (E) Representative confocal images of motor neurons from *Sacs^−/−^* (Sacs KO) or WT mice immunolabelled for ubiquitin. **(**F**)** Quantification of the number of motor neurons (MN) showing a perinuclear localization of ubiquitin. (**G**) Representative confocal images of sensory neurons from *Sacs^−/−^* (Sacs KO) or WT mice immunolabelled for ubiquitin. (H**G** Quantification of the number of sensory neurons (SN) showing a perinuclear localization of ubiquitin. Arrows show areas of ubiquitin accumulation. A white asterisk indicates the location of a glial cell. Scale bars =10 µm. Error bars are ±SD, *P < 0.05. *N*= between 30 and 70 neurons per experiment.

We previously reported abnormal bundling of neurofilaments in primary spinal motor and sensory neurons cultured from *Sacs*^−^^/^^−^ mouse embryos (8). In 4-week-old dissociated spinal cord cultures, bundling of neurofilaments was detected in the soma of *Sacs*^−^^/^^−^ motor (85.4 ± 4.9%) and sensory neurons (73.5 ± 8.5%) by immunolabelling with antibody against the high molecular weight neurofilament protein (NFH), as previously described (8) (Fig. 7A). In motor neurons, the normal neurofilament network courses through the cell body into the dendritic processes, whereas DRG sensory neurons lack dendrites. In *Sacs*^−^^/^^−^ motor neurons, neurofilaments coalesce into linear bundles, whereas in DRG neurons, they can form a juxtanuclear ball reminiscent of vimentin bundles in fibroblasts. Peripheral displacement of the nucleus was particularly visible in DRG sensory neurons with a collapsed neurofilament network (Fig. 7B and C). Mitochondria were excluded from the region of the neurofilament accumulations (Fig. 7D). Moreover, ubiquitin had a more perinuclear distribution in *Sacs*^−^^/^^−^ neurons compared with controls (Fig. 7G and H); this was statistically significant in both motor neurons (*P* < 0.05) and DRG sensory neurons (*P* < 0.05) (Fig. 7E and H). In contrast to ARSACS patient HDFs, there was no significant redistribution of the autophagy–lysosomal proteins Lamp2 or p62 in *Sacs*^−^^/^^−^ neurons (not shown).

## Discussion

Here, we present evidence that organization of the vimentin cytoskeleton is dramatically altered in ARSACS HDFs with a range of sacsin mutations and in cellular models where sacsin has been depleted. This is in agreement with our previous findings that abnormal accumulations of neurofilament occur in *Sacs*^−^^/^^−^ primary neurons (8). Importantly, these data indicate that loss of sacsin impacts on more than one type of intermediate filament, suggesting sacsin function may be broadly required for normal intermediate filament organization.

Cells lacking functional sacsin also exhibit abnormalities in organelle distribution, such as fragmentation of the Golgi in ARSACS HDFs and asymmetrical positioning of nuclei in *Sacs*^−^^/^^−^ neurons. This is consistent with intermediate filaments playing a role in intracellular organelle distribution (29,30) and may be relevant to the pathogenesis of ARSACS. We have previously shown recruitment of the fission factor Drp1 to mitochondria is impaired in sacsin null cells, proposing this partly explains the alterations in mitochondrial dynamics and function observed in cellular models of ARSACS (9). Displacement of mitochondria at the periphery of vimentin accumulations in ARSACS HDFs suggests that disrupted organelle distribution may also impact mitochondrial network organization and potentially function. Interestingly, in HDFs transiently transfected with siRNA targeting sacsin, there was not an obvious alteration in mitochondrial localization compared with that which we observed in ARSACS patient HDFs. We speculate that this difference is likely due to partial knockdown of sacsin and/or that the time after transfection (48 h) was not sufficient to induce this aspect of the loss of sacsin phenotype.

Collapse of the vimentin network around the MTOC occurs when mechanisms regulating intermediate filaments are disrupted and as well as in response to stress conditions such as the accumulation of misfolded protein aggregates (18,23,31). We did not see any direct evidence for accumulation of protein aggregates in ARSACS patient HDFs by TEM, or find the UPS to be compromised (i.e. no accumulation of ubiquitinated proteins). This suggests that the altered vimentin network in sacsin deficient cells is not the result of a problem with clearance of ubiquitinated proteins. Although ubiquitinated proteins did not accumulate in ARSACS HDFs, multiple components of the proteostasis machinery were relocalized to the pericentriolar region of vimentin accumulation. These included HSP70 and components of the autophagy–lysosome machinery. Interestingly, levels of p62 were decreased in ARSACS fibroblasts, while Lamp2 levels were elevated. p62 is degraded by autophagy and Lamp2 promotes fusion of autophagosomes with lysosomes (28,32). We analysed LC3 levels to determine if autophagy was activated in ARSACS HDFs, demonstrating autophagic flux was increased under conditions of nutrient starvation. These data suggest that the relocalization of lysosomes in ARSACS HDFs is associated with functional changes in the autophagy–lysosome pathways. This would be in agreement with juxtanuclear clustering of lysosomes being associated with increased autophagosome–lysosome fusion rates (33,34).

The phenotypes observed in ARSACS HDF and primary neurons from the *Sacs*^−^^/^^−^ mouse only partially recapitulated each other. For example, while vimentin is organized as a ‘cage’ in some ARSACS HDFs, neurofilaments form bundles, especially in dendrites, reflecting a difference in the organization of the cytoarchitecture. Moreover, we did not see any evidence for relocalization of components of the autophagy–lysosomes pathway in primary neurons from the *Sacs*^−^^/^^−^ mouse, but did see perinuclear accumulation of ubiquitin in neurons from the *Sacs*^−^^/^^−^ mouse. This ubiquitin staining may represent ubiquitinated proteins that are destined for removal by the autophagy–lysosome pathway, although we did not see any evidence for relocalization of lysosomes or autophagy proteins in primary neurons. These differences between neurons and fibroblasts may reflect cell-specific aspects of cytoskeleton or proteostasis regulation. Indeed, previous reports show that common inducers of autophagy in non-neuronal cells fail to stimulate autophagy in primary neurons, highlighting that autophagy in neurons is regulated by mechanisms that may differ from those in non-neuronal cells (35,36).

The sequence of events that lead to neuronal cell death in ARSACS is unknown but most of the evidence supports that the cytoskeletal disorganization is an early phenomenon (8). Altered proteostasis, mitochondrial dysfunction and cytoskeletal abnormalities are all features observed in other neurodegenerative diseases. These components of molecular pathology frequently occur in combination with multiple complex links between them. For ARSACS, based on the presence of domains linking to molecular chaperones and the UPS it would be reasonable to speculate sacsin has a role in protein quality control, most likely for a specific client or family of client proteins. If this is the case it would suggest that loss of sacsin function would result in altered levels or function of these clients at an early stage in the molecular pathogenesis of the disease. Our data may suggest that these clients will be linked to proteins involved in regulation of intermediate filaments or could even be intermediate filament proteins.

In conclusion, this study shows that loss of sacsin effects organization of intermediate filament networks with consequences for intracellular architecture. In fibroblasts, these include repositioning of components of the autophagy–lysosome pathway and subsequent alterations of autophagic activity.

## Materials and Methods

### Cell culture and sacsin knockdown

ARSACS patient fibroblasts were a gift from Dr Sascha Vermeer at Radboud University Nijmegen Medical Centre (Nijmegen, The Netherlands) and Dr Paola Giunti at UCL Institute of Neurology (London, UK). The cells were collected as part of a project approved by the Medical Ethics Committee of the Radboud University (CMO-nr 2014/155), and Giunti has ethical approval for dermal fibroblast collection through a European integrated project on spinocerebellar ataxias (REC Ref - 04/Q0505/21). Written informed consent to participate in this study was obtained from all patients. Control and ARSACS HDF lines used in this work were not closely age or sex matched, but were all between passage 3 and 8. HDFs were cultured in Dulbecco’s Minimum Eagle Medium (DMEM) supplemented with 10% foetal bovine serum (FBS) and 50 U/ml penicillin and 50 μg ml^−^^1^ streptomycin (final concentration in media 1%). All cells were kept in a constant humidified atmosphere of 5% CO_2_ at 37°C. Cell culture reagents were from Life Technologies (Paisley, UK).

SH-SY5Y cells were from the American Type Culture Collection and were grown in DMEM at a 1:1 ratio with Ham’s F12 medium. Cells were maintained in medium supplemented with 10% heat-inactivated FBS containing 100 U ml^−^^1^ penicillin and 100 mg ml^−^^1^ streptomycin.

For sacsin knockdown, a combination of three previously validated siRNAs targeting exons 7 (sense: GGAUGAUCCUCUGAAGGUC), 8 (sense: GCGGCCGAAUUCUAUAAAG) and 10 (sense: CGUAAGAUUUCUAGAUGAC) of *SACS* were used (2,4). These siRNAs were at a concentration of 10 nM each and were transfected in combination using Lipofectamine 3000 (ThermoFisher Scientific, UK), according to the manufacturer’s instructions. A negative control siRNA that has no significant sequence similarity to human gene sequences was used as a control at a concentration of 30 nM.

Generation of CRISPR/Cas9 *SACS*^−^^/^^−^ Flp-In T-REx HEK293 cells was performed following the manufacturers protocol for the sacsin double nickase plasmid (SantaCruz Biotechnology, cat. no. sc-404592-NIC). For inhibition of the proteasome MG132 was added to cells at a final concentration of 10 µm for 3 h. For inhibition of autophagy, Bafilomycin was added to cells at a concentration of 100 nm for 3 h and 3-MA at a concentration of 5 mM for 3 h.

*Sacs* ^−^^/^^−^ primary neurons were cultured from the *Sacs*^−^^/^^−^ mouse as described previously (2,8).

### Immunofluorescent detection and staining

Immunofluorescent labelling was as described previously (2). In brief, cells cultured on glass coverslips were fixed with 4% formaldehyde for 15 min and then permeablized for 5 min with 0.2% Triton-X 100. Cells were incubated with primary antibodies for 2 h in 0.02% Triton-X100, 1% bovine serum albumin (BSA) and 10% normal goat serum, prior to washing and incubation with fluorescently labelled secondary antibodies [Alexa Fluor 488-conjugated goat anti-rabbit or anti-mouse, Alexa Fluor 543 conjugated goat anti-mouse or anti-rabbit, or Alexa Fluor 647-conjugated goat anti-mouse or anti-rabbit (ThermoFisher Scientific)]. Cells were then counterstained with DAPI and coverslips mounted for microscopy. Primary antibodies were used at the following titres; 1:100 for rabbit monoclonal anti-vimentin (Abcam, UK), 1:100 for mouse monoclonal anti-vimentin (Abcam), 1:100 for rabbit monoclonal anti-sacsin (Abcam), 1:500 for mouse monoclonal anti-GS28 (Enzo Life Sciences, UK), 1:100 for mouse monoclonal anti-EEA1 (BD Biosciences, UK), 1:100 for mouse monoclonal anti-HSP70 (Sigma, Poole, UK), 1:100 for rabbit polyclonal anti-ubiquitin (Abcam), 1:100 for mouse monoclonal anti-Lamp2 (Santa Cruz, USA), 1:100 for mouse monoclonal anti-p62 (Abcam) and 1:100 for rabbit polyclonal anti-LC3 (Abcam). For staining of mitochondria with MitoTracker (ThermoFisher Scientific) the stock solution was diluted to a concentration of 100 nM in cell culture media prior to addition to cells for 30 min at 37°C in 5% CO_2_ atmosphere. After the incubation period, cells were washed twice in cell culture media prior to live imaging or fixation. Confocal microscopy was performed using a LSM510 or an LSM880 (Zeiss) with a 63× objective. Quantification of incidence of cells with perinuclear localization of HSP70, ubiquitin, Lamp2 and p62 was performed blind to experimental status. Incidence of LC3 punctae were quantified from confocal Z-stacks using a combination of the Surpass module and MeasurementPro modules of Imaris (Imaris 7.6.1 Bitplane, Concord, USA). Surface rendered 3D images were generated, with thresholding for punctae size and intensity consistent between images and experimental conditions.

### Immunoblotting

Proteins were size-fractionated using precast 4–12% gradient NuPAGE Bis-Tris gels (Life Technologies) and transferred to nitrocellulose membranes (Whatman). Membranes were blocked in either 5% (w/v) non-fat milk powder or 5% (w/v) BSA before probing with the specified antibodies. Primary antibodies were used at the following titres; 1:1600 for rabbit monoclonal anti-sacsin (Abcam), 1:5000 for mouse monoclonal anti-HSP70 (Sigma) 1:5000 for mouse monoclonal anti-Lamp2 (Santa Cruz), 1:3000 for rabbit polyclonal anti LC3 (Abcam) 1:10 000 for mouse monoclonal anti-β-actin (Sigma), 1:5000 for rabbit polyclonal anti-GAPDH (Abcam), 1:1000 for mouse monoclonal anti-p62 (Abcam), and 1:3000 for rabbit polyclonal anti-LC3 (company). Immunoreactive products were visualized and quantified, after labelling with species-specific infrared secondary antibodies (1:5000, LI-COR Biosciences, UK), using the Odyssey imaging system (LI-COR). Apparent molecular masses were estimated using the Novex Sharp Pre-stained Protein Standard (Life Technologies) or HiMark Pre-stained Protein Standard (Life Technologies) and the band sizing application in Odyssey software (LI-COR Biosciences). Images of the immunoblots were also quantified using the Odyssey software.

### Transmission electron microscopy

Cells were grown on glass or Thermanox cover-slips and fixed overnight in cold (4°C) Karnovky’s fixative (2% paraformaldehyde and 2.5% glutaraldehyde in 0.08M cacodylate buffer). They were then washed three times in phosphate buffer and osmicated with 1% osmium tetroxide in ddH_2_O for 1 h. Samples were then washed 3×10 min in ddH_2_O and dehydrated with a series of alcohols: 30, 50, 70, 90, 3×100% and 2× propylene oxide (at least 20 min in each). They were infiltrated with 50% propylene oxide: 50% araldite resin overnight and with several changes of 100% resin the next day. Resin blocks were cured at 60°C overnight. Sectioning was done using a Leica Ultracut UCT microtome. Sections were counter-stained Reynold’s lead citrate and were viewed on a JEOL 1010 TEM (JEOLUSA, USA).

### Statistical analyses

All data are expressed as means ± SEM, unless otherwise stated. Statistical significances were determined using Mann–Whitney *U* tests or unpaired Student’s *t*-tests as appropriate.

## Supplementary Material

Supplementary Material is available at *HMG* online.

## Supplementary Material

Supplementary Figures
