# Supplementary material for: Altered organization of the intermediate filament cytoskeleton and relocalization of proteostasis modulators in cells lacking the ataxia protein sacsin
Source: Hum Mol Genet. 2017 May 23;26(16):3130–43. doi: 10.1093/hmg/ddx197 (PMC5886247; doi:10.1093/hmg/ddx197)
Supplement: Supplementary Figures [file hmg-2017-d-00364_supplemental_ddx197.pdf]

A

ARSACS patient *SACS* mutations#	Type of mutation	Sacsin detected by immunoblot
p.Q4054*& c.2094-2A>G	Nonsense/Splice-site	No
p.R2002fs & p.Q4054*	Frameshift/Nonsense	No
p.F2780C & p.S3892*	Missense/Nonsense	No
p.2801delQ	In frame deletion	Yes
p.K1715* & p.R4331Q	Nonsense/Missense	?
p.R3636Q : p.P3652T & p.L3745Rfs	Missense/Frameshift	No
p.R3636Q : p.P3652T & p.C72Cfs	Missense/Frameshift	Yes
c.8844delT	Nonsense	No

SACS Accession number NP_055178

Top five listed cell lines were predominantly used in this study

B

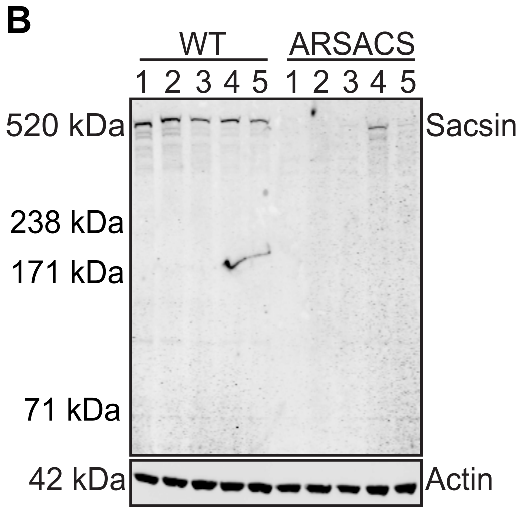

ARSACS lines
1 = Q4054* & c.2094-2A>G
2 = R2002fs & Q4054*
3 = F2780C & S3892*
4 = p.2801delGln
5 = K1715* & R4331Q

C

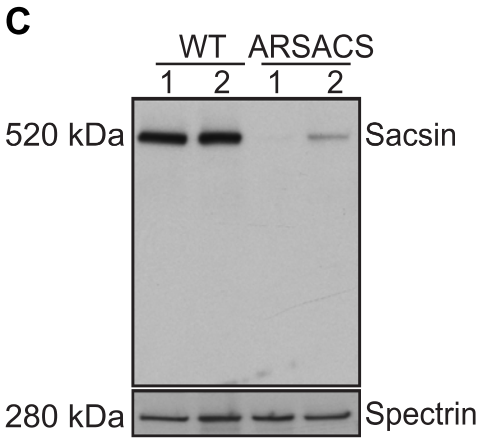

ARSACS lines
1 = R3636Q : P3652T & L3745Rfs
2 = R3636Q : P3652T & C72Cfs

D

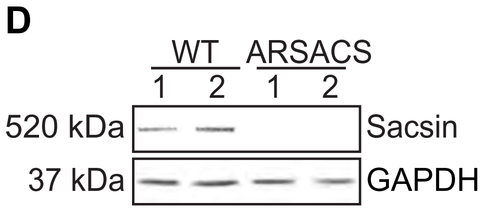

ARSACS lines
1 & 2 = c.8844delT

Figure S1. ARSACS HDF lines used in this study. (A) Table detailing the disease associated mutations present in sacsin for each ARSACS patient cell line used in this study. Amino acid change to an * indicates a truncated protein, fs indicates a frameshift and del indicates a deletion. For the column 'sacsin detection by immunoblot' it should be noted that the anti-sacsin antibody available recognises an epitope between amino acids 4100-4200 of sacsin. **(B)** Immunoblot to detect sacsin in total cell lysates from wild-type control and ARSACS HDFs with the mutations p.Q4054*/c.2094-2A>G, p.R2002fs/p.Q4054*, p.F2780C/p.S3892*, p.2801delGln, and p.K1715*/p.R4331Q. β-actin was used as a loading control. **(C-D)** Immunoblot to detect sacsin in cell lysates in additional ARSACS HDFs with the mutations p.R3636Q/p.P3652T/p.L3745Rfs, R3636Q/P3652T/C72Cfs, and c.8844delT.

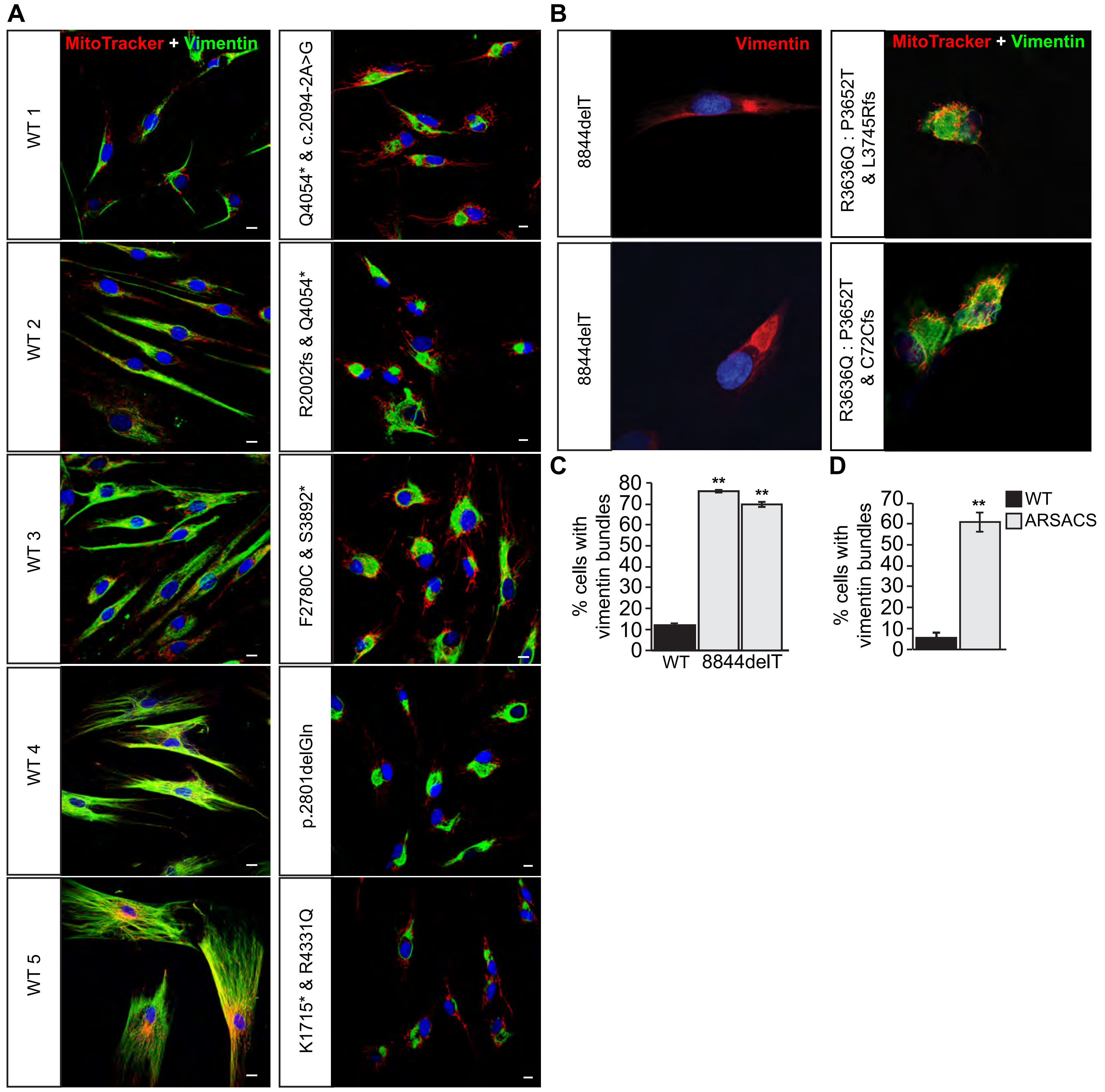

Figure S2. Abnormal accumulations of vimentin intermediate filament are present in HDFs from ARSACS patients with a range of different mutations. (A) Representative confocal images of five ARSACS patient HDFs and five wild-type (WT) control HDF lines that were stained for mitochondria (MitoTracker) and immunolabeled for vimentin. Cells were also stained with DAPI to detect nuclei. Scale bars = 10 µm. **(B)** Analyses of vimentin localisation in additional ARSACS HDFs with the mutations c.8844delT, p.R3636Q/p.P3652T/p.L3745Rfs, and p.R3636Q/p.P3652T/p.C72Cfs. **(C)** The percentage of cells with a collapsed vimentin network was quantified in the two patient cell lines that are homozygous for the c.8844delT mutation. This was done blind to experimental status with >120 cells scored per cell line. Results are expressed as mean ± SEM for wild-type and patient cell lines. **(D)** The percentage of cells with a collapsed vimentin network was quantified in the ARSACS HDFs with the mutations p.R3636Q/p.P3652T/p.L3745Rfs and p.R3636Q/p.P3652T/p.C72Cfs. This was done blind to experimental status with >120 cells scored per cell line. Results are expressed as mean ± SEM for wild-type and patient cell lines. **p<0.01.

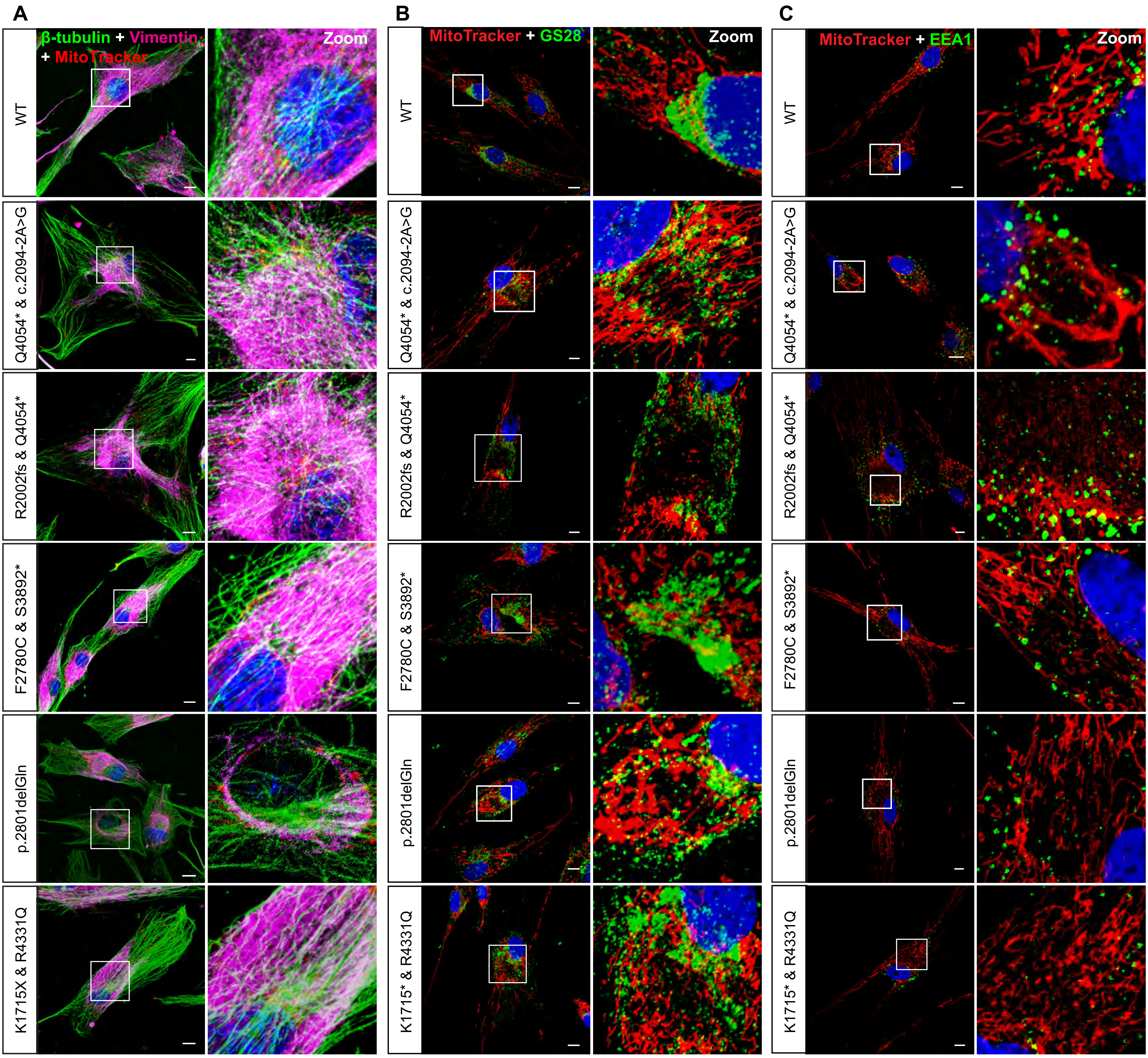

Figure S3. ARSACS patient HDFs have accumulations of vimentin in the region of the MTOC as well as altered localisation of the Golgi apparatus and early endosomes. (A-C) Representative confocal images of five ARSACS patient HDFs lines and a wild-type (WT) control. Cell were stained with MitoTracker before being processed for immunofluorescent detection of (A) β-tubulin and vimentin, **(B)** GS28 membrane protein of the cis-Golgi, or **(C)** EEA1, membrane-associated protein to early endosomes. Cells were counterstained with DAPI for nuclei. White boxes are shown as zoomed images in the right-hand panels. Scale bars = 10μm.

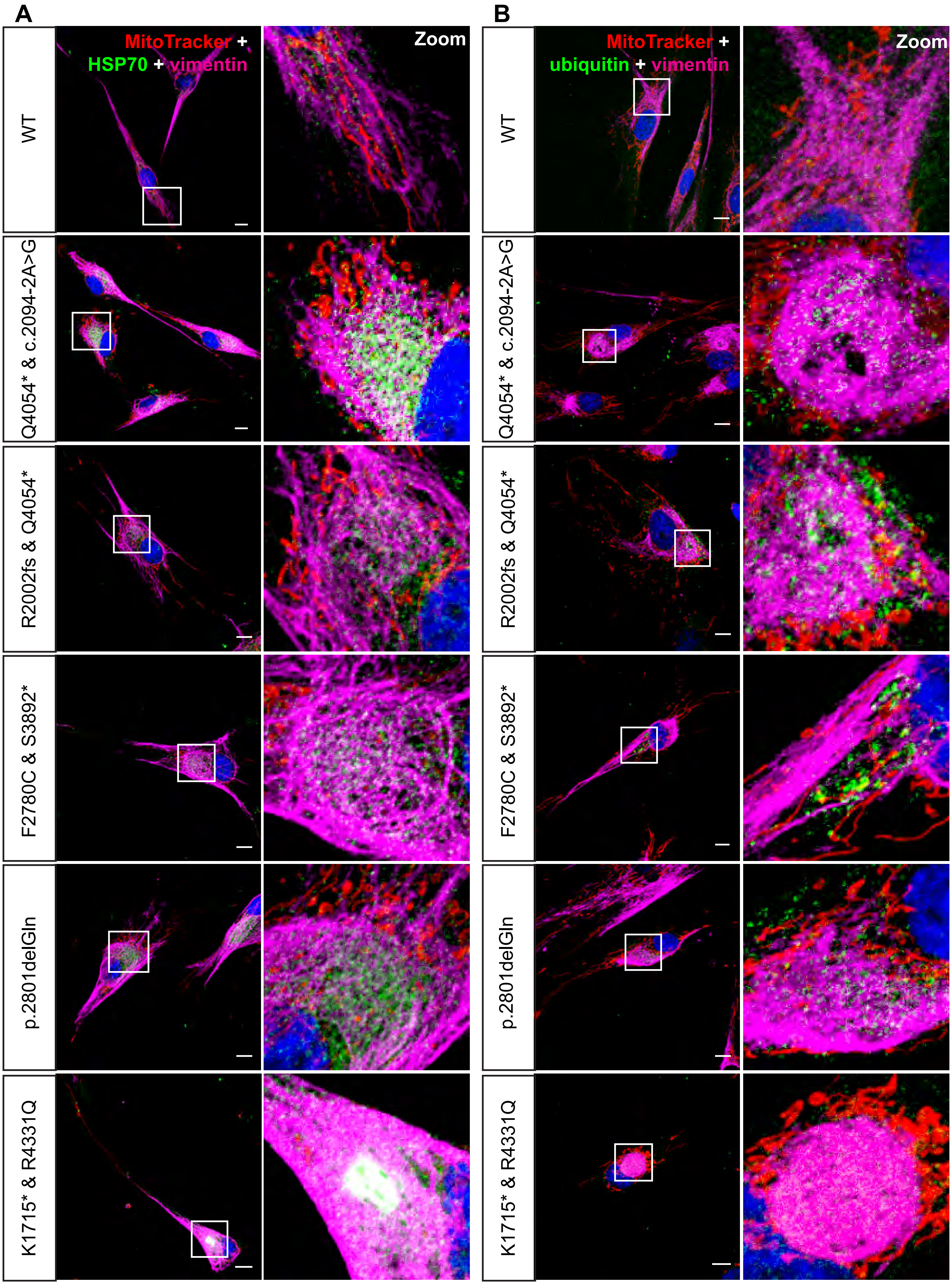

Figure S4. ARSACS patient HDFs localise components of the cellular proteostasis machinery to a vimentin cage structure. (A, B) Representative confocal images of dermal fibroblasts from ARSACS patient or wild-type (WT) control. Cells were stained with 100μM MitoTracker for 30 minutes prior to fixation and permeabilisation. Cells were incubated with antibodies to **(A)** HSP70 and vimentin, or **(B)** ubiquitin and vimentin. Confocal analysis showed accumulation of HSP70 and ubiquitin in perinuclear regions in all ARSACS patient cells. White boxes in merged panels are shown zoomed. Scale bars = 10 μm.

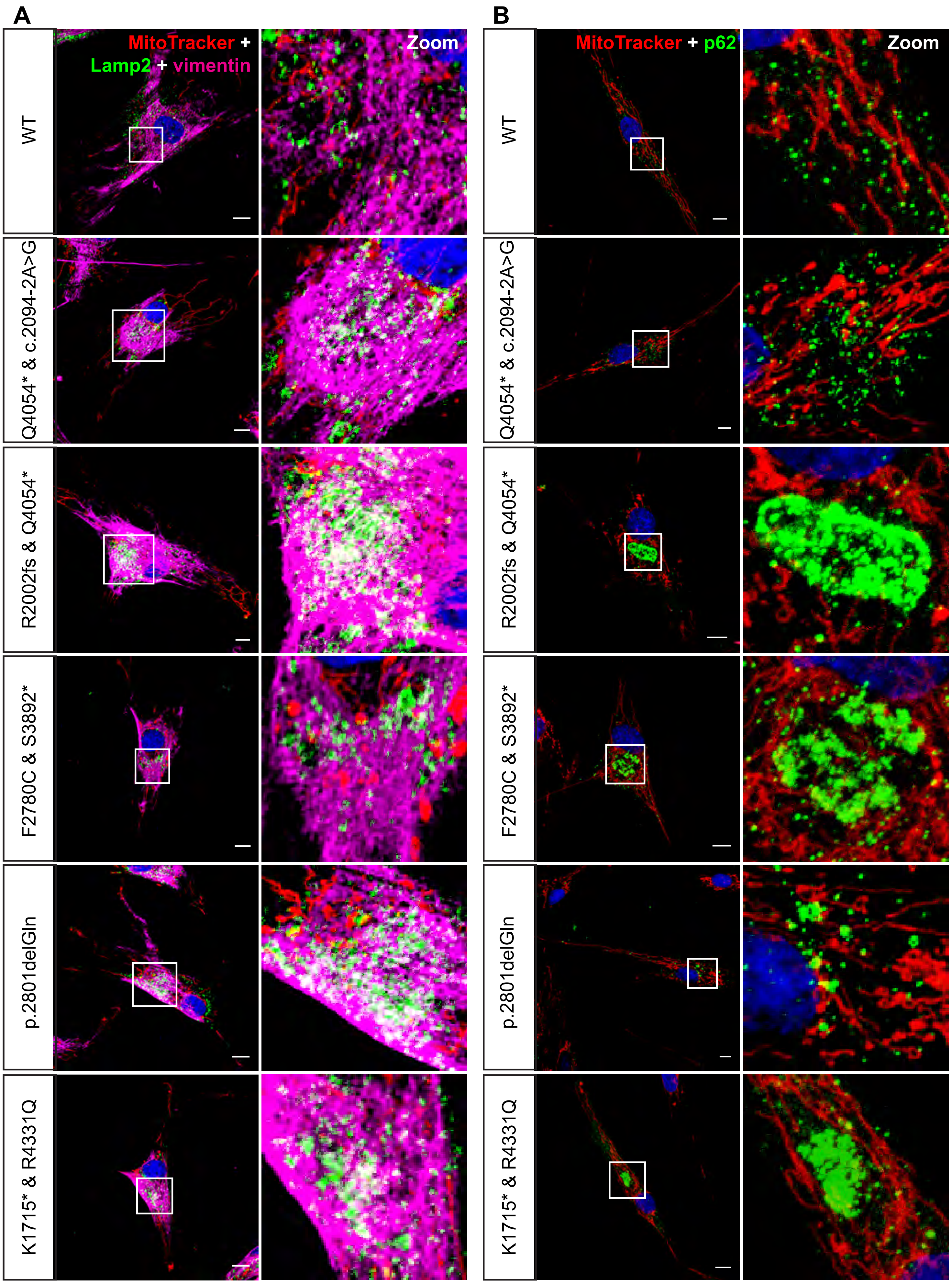

Figure S5. Accumulation of LAMP-2 and p62/SQSTM1 in ARSACS patient HDFs. (A) Representative confocal images of dermal fibroblasts from ARSACS patients or wild-type (WT) control. Cells were stained with 100μM MitoTracker for 30 minutes prior to fixation and permeabilisation. Cells were incubated with antibodies to **(A)** LAMP-2 and vimentin, or **(B)** p62/SQSTM1. Confocal analysis showed perinuclear accumulation of LAMP-2 and p62/SQSTM1 in ARSACS patient cells. White boxes in the merged panels are shown zoomed in the right-hand panels. Scale bars = 10 μm.
